# *EmoTIC*: Impact of a game-based social-emotional programme on adolescents

**DOI:** 10.1371/journal.pone.0250384

**Published:** 2021-04-16

**Authors:** Usue de la Barrera, Estefanía Mónaco, Silvia Postigo-Zegarra, José-Antonio Gil-Gómez, Inmaculada Montoya-Castilla

**Affiliations:** 1 Department of Personality, Assessment and Psychological Treatment, Faculty of Psychology, Universitat de València, Valencia, Spain; 2 Department of Psychology, Faculty of Health Sciences, Universidad Europea de Valencia, Valencia, Spain; 3 Instituto Universitario de Automática e Informática Industrial, Universitat Politècnica de València, Valencia, Spain; University of La Rioja, SPAIN

## Abstract

**Introduction:**

Technologies provide a brilliant opportunity to promote social-emotional competences, well-being and adjustment in adolescence. Game-based programmes and serious games are digital tools that pursue an educational goal in an attractive environment for adolescents. The purpose of this study was therefore to determine the effectiveness of *emoTIC*, a game-based social-emotional programme designed according to Mayer, Caruso, and Salovey’s model of emotional intelligence.

**Materials and methods:**

The participants were 119 adolescents between 11 and 15 years, randomly assigned to the experimental group and the control group. The adolescents completed questionnaires to assess their emotional intelligence, self-esteem, affect balance, difficulties, prosocial behaviour, depression, anxiety and stress.

**Results:**

The MANCOVA results showed that adolescents who completed the game-based programme had improved self-esteem, affect balance, emotional symptoms, behavioural problems, and hyperactivity (Wilks’ λ = .77; *F* = 2.10; *p* = .035). Hierarchical multiple regression indicated that adolescents in the experimental group had a greater change in self-esteem and affect balance (positive β), while their emotional problems and hyperactivity decreased (negative β). Anxiety moderated the influence of the intervention on self-esteem (*b* = .04; *t* = -2.55; *p* ≤ .05; LLCI = -0.43, ULCI = -0.05). Adolescents with low or medium anxiety improved their self-esteem with the intervention, while those with high anxiety did not develop it.

**Conclusions:**

The use of technology in social-emotional programmes could be the first step in increasing adolescents’ interest in emotions and emoTIC could be considered a useful programme which influences their personal, emotional and social factors.

**Trial registration:**

Clinical Trial identifier: NCT04414449.

## Introduction

### Game-based learning and serious games for adolescents

The last two decades have seen the rapid ascent of computing technology to change health behaviour and well-being [1]. As today’s adolescents are strongly attracted to and familiar with the use of technologies, these are a brilliant opportunity to promote learning and well-being in this stage of development [2]. In addition to technologies, gamification to turn intervention programmes into digital games is considered a good way to intrinsically motivate adolescents to engage with their use [3]. Games are systems purpose-built for motivation through their capacity for enjoyment [4] as positive emotions may facilitate learning and contribute to academic achievement [5]. Digital game-based learning refers to using the entertaining power of digital games for an educational purpose, using a balance between learning and gaming elements [6]. Serious games are digital tools to facilitate skills or knowledge acquisition in an entertaining environment, and integrate both pedagogical theories and the motivational principles of game design in pursuit of an educational goal [7–10].

### Emotional intelligence, self-esteem and social-emotional intervention programmes in adolescents

Adolescence is a complex stage of the life development with numerous emotional, social and physical changes that may have a negative impact on health [11]. Mood disorders such as depression or anxiety have a high risk of emerging and developing during this stage [12, 13]. Promoting strengths during adolescence is critical because adolescence is a sensitive period of brain development [14]. Emotional intelligence and self-esteem are two of the most important strengths studied in the social-emotional sphere. Emotional intelligence is conceptualised as a set of skills related to the perception, expression, and regulation of one’s own or others’ emotions as well as the use of those skills to achieve goals in life [15]. Self-esteem is considered as a favourable or unfavourable attitude that people have about themselves. During adolescence, both are positively associated with subjective well-being [16]. Concretely, individuals who perceive, know, and manage their emotions might deal better with emotional issues, and experience greater well-being [17]. Likewise, adolescents are particularly sensitive to the appearance of symptoms of emotional instability [18] and emotional competences and self-esteem are inversely related to emotional symptoms, behavioural problems, and peer conflicts [19, 20]. Specifically, adolescents who pay more attention to their emotional states combined with low emotional regulation and unfavourable attitude about themselves, experience more emotional symptoms. In the same way, adolescents with a poor emotional regulation and unfavourable attitude about themselves present more behavioural problems [16]. Education in social-emotional competences can therefore prevent the development of emotional and behavioural problems in adolescence [21, 22].

Social-emotional education is considered as important as the contents of the academic curriculum and school environments seem to be the most appropriate contexts in which to develop it [23]. Numerous programmes to improve social-emotional skills have been carried out in high schools with positive results [24–26]. Systematic reviews demonstrated the effectiveness of these programmes, most notably for improving emotional intelligence [27]. These programmes also have indirect positive consequences for the well-being and the peer relationships of their users [20, 28]. Although the benefits of intervention programmes in emotional education are evident, motivating adolescents through participation is a crucial challenge and games can be an effective educational tool to this purpose [29].

### Serious games and game-based social-emotional intervention programmes for adolescents

The existing literature has shown the effectiveness of digital game-based learning [30, 31]. Educational programmes for adolescents have been carried out using technologies to reduce school violence [32], prevent suicide [33], and encourage non-sexist attitudes [34], among other variables. The development of emotional intelligence carried out using technologies could be more attractive and useful for adolescents, and their commitment to the programme would increase [35, 36]. However, there is a gap of interventions that combine technologies and gamification to enhance adolescent social-emotional learning.

Few studies have evaluated the effectiveness of programmes to develop social-emotional competences in adolescents implemented using technologies. In the USA, Kahn et al. [37] designed the virtual game *RAGE-control* in order to improve emotional control for patients of the inpatient psychiatric service at Boston Children’s Hospital. The aim was to teach children and adolescents aged between 9 and 17 years to focus their attention and control their physiological arousal using relaxation and biofeedback techniques. In Thailand, Iaosanurak et al. [36] tested an intervention with elementary school-aged children to promote social-emotional learning based on technology. The intervention was effective in increasing emotional self-control. In Romania, David et al. [38] designed the online video game *REThink*, applied to coaching emotional understanding in adolescents aged between 10 and 16 years old. Their study aimed to evaluate the effectiveness of *Feeling Better*, a mini video which was game part of *REThink*, to teach children and adolescents to distinguish between functional and dysfunctional emotions. The participants’ ability to correctly identify and collect functional emotions increased after three trials of playing. In Spain, Cejudo et al. [39] developed the video game *Spock*, which provides training in emotional intelligence in adolescents aged 17 and 18 years old. The video game was effective in increasing emotional intelligence, adaptive skills and personal adjustment, and in reducing behavioural symptoms and externalizing problems. Another programme which has recently promoted social-emotional using Immersive Virtual Reality as a didactical tool was developed by Herrero and Lorenzo [40]. The results show a positive impact on the competences in children and adolescents from 8 to 15 years with autism spectrum disorders (ASD).

Although these social-emotional programmes showed their efficacy, there are several limitations. Some studies focused on non-healthy adolescents with mental disorders [37, 40], others included only older adolescents or young adults [39, 41], while others lacked a control group [36, 38] or evaluated the effectiveness of only one part of the intervention programme [38]. Likewise, none of the studies mentioned above discussed possible moderators of programmes’ effectiveness, such as demographic variables [42].

### The current study

These previous studies therefore reveal the need to develop social-emotional programmes involving technologies and test their effectiveness with healthy children and adolescents in the school context. As a result, the aim of the present study was to analyse the effectiveness of *emoTIC*, a game-based social-emotional programme for adolescents. The hypotheses were: (1) the programme will develop emotional intelligence and self-esteem; (2) the programme will have a positive effect on affect balance, emotional symptoms, behavioural problems, peer relationship problems, hyperactivity, and prosocial behaviour; (3) the demographic factors (gender and age) and affective symptoms (depression, anxiety, and stress) will moderate the effectiveness of the programme.

## Materials and methods

### Participants

**Fig 1:**
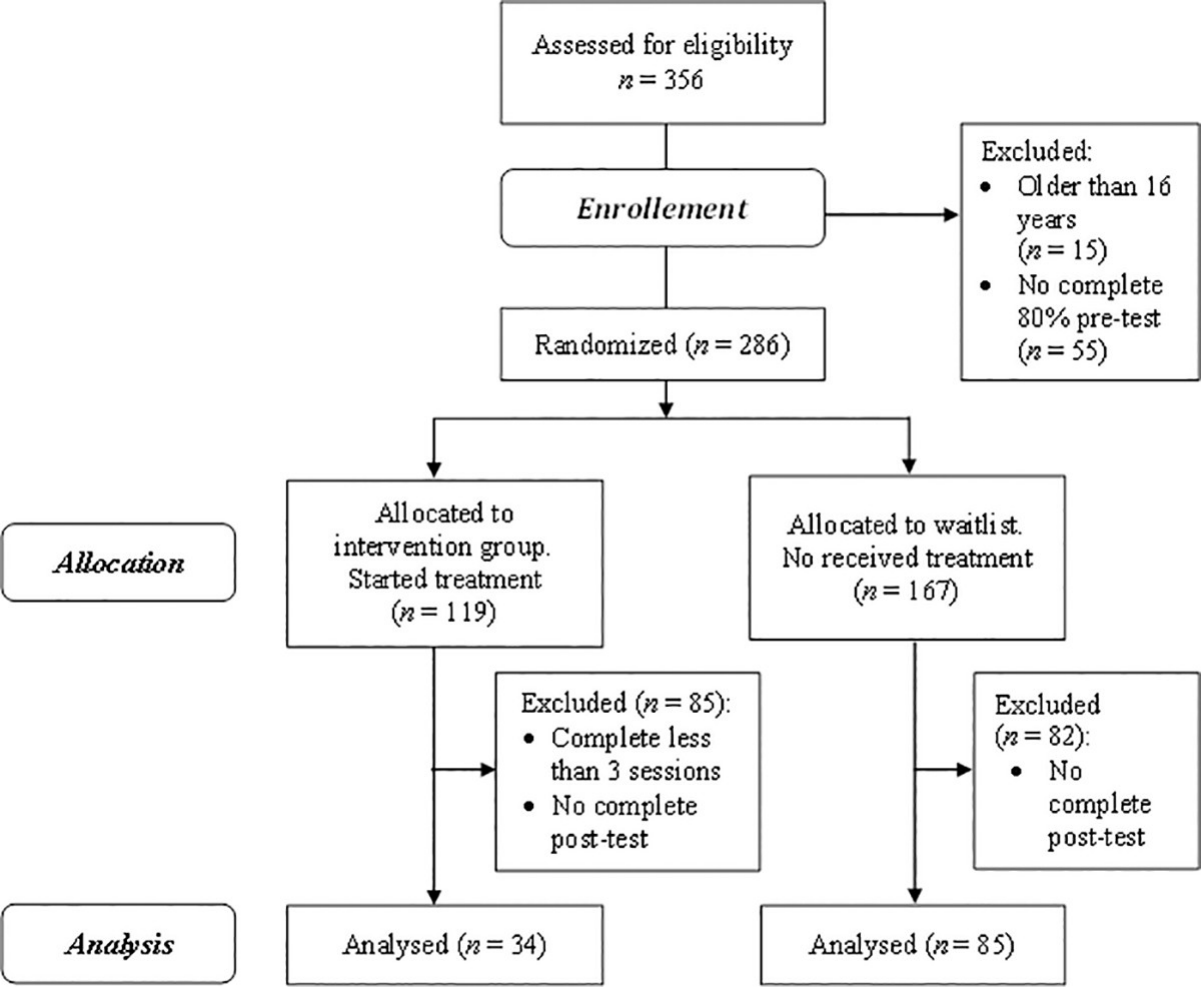
Flowchart of study participants and dropouts.

Initially, the participants were 356 adolescents of which 70 were eliminated due to exclusion criteria (Fig 1). The adolescents were randomized to an intervention group (*n* = 119) and a waiting list group (*n* = 167). Randomization was performed two weeks before the intervention programme commenced by a simple coin flip carried out by an external member of the research team. A total of 119 adolescents started the programme and 34 of them completed at least 3 sessions and post-test, while 167 did not received the intervention and 85 of them completed post-test. Finally, the participants were 119 adolescents aged between 11 and 15 years (*M* = 12.57 years; *SD* = 0.67; 42.9% girls). The adolescents were students from public and private high schools in Madrid (Spain). Approximately, 94% were of Spanish nationality and 6% were from other countries (Peru, Venezuela, Romania, and Equatorial Guinea), who had lived in Spain for between 2 and 12 years (*M* = 7.43; *SD* = 4.50). As regards their families, 94.1% reported that their nuclear family was composed of a mother and father, while 5.9% had a single-parent family. Adolescents born in other countries do not differ in emotional, social, personal, and family variables from adolescents born in Spain (*p* > .05; *d* < 0.45). As regards sexual orientation, 90.80% of adolescents were heterosexual, 0.80% were homosexual, 2.50% were bisexual and 5.90% were not sure about her or his sexual orientation. The inclusion criteria were to be enrolled in the first or second year of compulsory secondary education at a Spanish school, to have a mobile device, and to have informed consent signed by the parents. The exclusion criteria were being under 11 years old or over 16 years old and not complete at least 80% of the pre-test.

### Instruments

#### Sociodemographic variables

Gender, age, nationality, years in Spain, family type, and sexual orientation of the participants were assessed using *ad hoc* questions.

#### Emotional intelligence

Emotional intelligence is operationalised through emotional attention, emotional clarity, and emotional regulation. Emotional attention is the ability to feel and express feelings properly, emotional clarity is the ability to understand the own emotional states, and emotional repair is the capability to regulate emotional states properly. The Spanish version of the Trait Meta-Mood Scale-24 (TMMS-24) [43] validated in Spanish adolescents by Pedrosa et al. [44] was used. The instrument consists of 24 items, and the adolescents answer using a 5-point Likert scale (1 = *strongly disagree*, 5 = *strongly agree*). The scale is divided into three factors: attention (e.g., “I often think about my feelings”), clarity (e.g., “I almost always know exactly how I am feeling”), and repair (e.g., “If I find myself getting mad, I try to calm myself down”). The psychometric properties of the instrument were adequate in previous studies conducted with Spanish adolescents (α = .83 in the three dimensions) [45], as well as in this study (α between .81 and .83).

#### Self-esteem

From the one-dimensional perspective, self-esteem is considered a favourable or unfavourable attitude that adolescents have about themselves. The Rosenberg Self-esteem Scale (RSE) [46] was used. This instrument consists of 10 items (e.g., “I take a positive attitude toward myself”), scored on a 4-point Likert scale (1 = *strongly disagree*, 4 = *strongly agree*). Previous studies with Spanish adolescents showed adequate psychometric properties of the instrument (α = .87) [47]. The reliability index in our sample was adequate (α = .82).

#### Affect balance

The affective balance is the affective dimension of subjective well-being and it is operationalised through positive and negative affect experimented during the last month. It is the result of subtracting negative feelings score from the positive feelings score. The Scale of Positive and Negative Experiences (SPANE) [48] validated in Spanish adolescents [49] was used. The instrument is composed of 12 items with a 5-point Likert scale (1 = *very rarely or never*, 5 = *very often or always*), and the adolescents report how often they have experienced positive and negative affect during the last month. The scale is distributed into two factors: positive feelings (e.g., pleasant, joyful or good feelings) and negative feelings (e.g., unpleasant, afraid or bad feelings). The psychometric properties of the instrument were adequate in previous studies conducted with Spanish adolescents [50], as well as in this study (α between .81 and .87).

#### Emotional, behavioural and peer difficulties and prosocial behaviour

Emotional symptoms include having many worries, unhappiness, and many fears. Conduct problems include having hot tempers, fighting with other children, and lying. Peer problems include tends to play alone, bullied by others, and getting on better with adults than with other children. Hyperactivity includes being overactive, constantly fidgeting and easily distracted. Prosocial behaviour includes sharing with others, helping if someone is hurt or upset, and being kind with to younger children. The Strengths and Difficulties Questionnaire (SDQ) [51] validated in Spanish adolescents [52] was used to evaluate different emotional and behavioural problems and prosocial capacities related to mental health in adolescents. The scale is composed of 25 items, and the response format is a 3-point Likert scale (0 = *not true*, 2 = *certainly true*). The instrument is distributed in five subscales: (1) emotional symptoms (e.g., “I have many fears, I am easily scared”); (2) Conduct problems (e.g., “I take things that are not mine from home, school or elsewhere”); (3) Peer problems (e.g., “I am usually on my own. I generally play alone or keep to myself”); (4) Hyperactivity (e.g., “I am restless, I cannot stay still for long”); and (5) Prosocial behaviour (e.g. “I usually share with others (food, games, pens, etc.)”). The psychometric properties in previous studies conducted with Spanish adolescents were adequate (α between .72 and .87) [47], and studies suggest that the instrument may be an adequate tool for the screening of emotional and behavioural difficulties during adolescence [52]. The reliability index in the present study was sufficient (α between .50 and .68).

#### Depression, anxiety, and stress

Depression includes dysphoria, hopelessness, devaluation of life, self-deprecation, lack of interest, anhedonia, and inertia. Anxiety assesses autonomic arousal, skeletal muscle effects, situational anxiety, and subjective experience of anxious affect. Stress assesses difficulty relaxing, nervous arousal, and being easily upset, irritable and impatient. The Depression, Anxiety, and Stress Scales (DASS-21) [53] Spanish adaptation [54] was used to assess the affective symptoms during the previous week. The scale is composed of 21 items with a 4-point Likert scale (0 = *never*, 3 = *almost always*). The instrument evaluates three subscales: depression (e.g., “I was unable to become enthusiastic about anything”), anxiety (e.g., “I felt scared without any good reason”), and stress (e.g. “I found it difficult to relax”). Previous studies indicated adequate psychometric properties in Spanish samples (α between .73 and .81) [55], as well as in the present study (α between .73 and .80).

### Intervention programme

The game-based programme called *emoTIC* focuses on developing social-emotional competences in adolescents (Tables 1 and 2). Studies suggest that positive emotions may facilitate learning and contribute to academic achievement [5]. For that reason, the programme was designed as a space adventure, and the adolescents had to rebuild a spaceship to return to Earth. The digital application could be used on a smartphone or tablet, and is available for iOS and Android. *EmoTIC* is based on the ability model of emotional intelligence developed by Mayer and Salovey [56, 57]. According to the model, emotional intelligence is explained by four hierarchical components: (1) perception, evaluation, and expression of emotion; (2) emotion as a thought facilitator; (3) understanding emotions; and (4) management of emotions. In addition to emotional intelligence, self-esteem, group cohesion, and positive classroom climate were enhanced across the board. The programme consists of four classroom group sessions and twelve individual home activities. Each of the classroom group sessions followed the same structure. First, each adolescent performed an emotional self-examination to detect how he or she was feeling and shared it with the group. Second, a group dynamic was carried out according to the topic of the session (Table 2), followed by a discussion and group reflection. Finally, a summary of the session was made by the teacher. As regards individual home activities, adolescents completed three activities per area. Of the three activities, the first one was dedicated to the acquisition of basic concepts, the second one focused on the improvement of self-knowledge, and the last one tested their skills. A previous mixed model study (qualitative and quantitative) showed that adolescents who used *emoTIC*, perceived to have improved their emotional competences, social skills, and personal strengths [58].

**Table 1:** Sessions, activity code, title of activities, general purpose, and topics in the programme.

Session	Code^a^	Title of activity	General purpose	Topics
1	1.1	What are emotions?	To promote group cohesion and develop perception, evaluation and expression of emotions.	• Perception, evaluation and expression of emotions • Group cohesion and positive classroom climate • Basic concepts related to emotions
	1.2	Who is feeling the emotion?		
	1.3	DiMOOD I		
	1.4	Hidden faces		
2	2.5	Emotions and thoughts	To understand the relationship between emotions and thoughts.	• Facilitating our thought using our emotions • Our decision-making is affected by our emotional states • Taking perspective of and empathize with others
	2.6	Intelligent optimism		
	2.7	DIMOOD II		
	2.8	What is going on?		
3	3.9	Understanding my emotions	To understand emotions and promote self-esteem	• Understanding emotions and using emotional knowledge • Labelling emotions • Promoting self-esteem
	3.10	Focusing		
	3.11	Emotional network		
	3.12	Your own strengths		
4	4.13	How can I manage my emotions?	To develop emotional regulation	• Emotional regulation to promote emotional and intellectual growth • Emotional regulation strategies • Resolving conflicting emotional situations
	4.14	Traffic lights		
	4.15	Emotional strategies		
	4.16	You got a message!		

^a^First digit corresponds to the session and the second digit corresponds to the activity.

**Table 2:** Sessions, type of activity, goals, and activity content of the programme.

Code^a^	TA^b^	Goals	Activity content
1.1	GA^c^	To acquire basic concepts about emotions and promote positive climate	Adolescents discuss and elaborate in groups a definition for the concept of emotion. EmoTIC provides words and feedback.
1.2	IA^d^	To identify the facial expression of basic emotions	A name of an emotion and a set of people’s faces are presented on the smartphone. Adolescents select faces that express that emotion.
1.3	IA^d^	To understand the two main dimensions of emotions: arousal and hedonic valence.	Adolescents indicate on the smartphone their own perception of the level of arousal and hedonic valence of each basic emotion.
1.4	IA^d^	To identify the facial expression of basic emotions	The black stripes covering the faces gradually disappear and adolescents have to identify the emotion as soon as possible.
2.5	GA^c^	To experience the influence of emotions on thoughts.	An activity to show the learned helplessness is proposed on the smartphone. Adolescents then discuss the influence of emotions on thoughts.
2.6	IA^d^	Analyse emotions and thoughts that emerge in emotional situations.	Adolescents analyse a variety of emotional situations, try to understand feelings and propose the intelligent optimist thought. EmoTIC provides feedback.
2.7	IA^d^	To deepen in the two main dimensions of emotions: arousal and hedonic valence.	Adolescents indicate their perception of the arousal and hedonic valence levels in complex emotions. Smartphones provide feedback.
2.8	IA^d^	To take different perspectives and understand how what we think influences how we feel.	An emotional situation is presented on the smartphone. Adolescents try to understand how the person feels based on the thoughts that the situation generates in the characters.
3.9	GA^c^	To understand the role and the information of each emotion.	Adolescents listen to an audio about feelings using their smartphones. They discuss the role of emotions and the information provided by moods.
3.10	IA^d^	To understand one’s own emotions, to label them and to appreciate their relevance.	Adolescents focus on their feelings at that moment through smartphone-guided audio. They try to draw and label their emotions.
3.11	IA^d^	To learn about the families of emotions	Adolescents classify different emotions by families using their smartphones.
3.12	IA^d^	To promote strengths and self-esteem	Adolescents reflect on their strengths and try to solve an emotional problem offered by emoTIC.
4.13	GA^c^	To know some strategies of emotional regulation.	Adolescents role-play emotional situations using different regulation strategies randomly selected by their smartphone.
4.14	IA^d^	To increase knowledge about emotional regulation	Adolescents use a traffic light to indicate their agreement or disagreement with affirmations about emotional regulation. EmoTIC provides feedback.
4.15	IA^d^	To increase knowledge about emotional regulation strategies	Adolescents choose the most appropriate emotional regulation strategy for each of the conflicting emotional situations they face.
4.16	IA^d^	To analyse the emotional process	A conflict situation is proposed by smartphones. Adolescents answer the questions through messages as a conversation with a friend.

^a^Code of activity according to Table 1.

^b^Type of activity.

^c^Group activity.

^d^Individual activity.

### Procedure

The study was approved by the Ethics Commission of the University of Valencia (H152865096049), and we followed the standards of the Declaration of Helsinki to collect the data. Convenience sampling method was used to recruit participants. First, the principal investigator contacted the school principals and explained the objectives of the research. The parents who agreed to have their children participate signed the informed consent. The adolescents were randomized to an experimental group and a control group. The teachers completed a training course to learn about using *emoTIC* and implemented the intervention programme in their tutorial classes. The experimental group and the control group were initially evaluated. First, socio-demographic data were collected and each student provided a random participation code. To avoid fatigue and the development of negative feelings, questionnaires assessing negative variables (e.g., depression, anxiety and stress symptoms) were alternated with those assessing positive variables (e.g., life satisfaction). The duration of the assessment of adolescents did not exceed 50 minutes. The experimental group then carried out the programme during four weeks, while the control group continued with the usual tutorial classes. The intervention programme was conducted using a smartphone or tablet. After the implementation of the programme, both groups were evaluated again.

### Data analysis

First, the effectiveness of the programme was analysed by a multivariate analysis of variance (MANOVA) and a multivariate analysis of covariance (MANCOVA) using SPSS version 23. The MANOVA was performed to identify possible differences at T1 between the control group and the experimental group. The MANCOVA was performed in order to identify changes at T2, controlling for T1 scores (covariable). Power analysis were performed using G*Power 3.1.9.7. [59]. Second, multiple hierarchical regression analyses were carried out to examine the impact of the intervention programme. The dependent variables were the change in emotional intelligence, self-esteem, affect balance, difficulties, and prosocial behaviour between T1 and T2. In the first step, the T1 score (control variable) was introduced in the model. The independent variable entered in the second step was the experimental condition (0 = *control group*; 1 = *experimental group*). Third, the reliable change index (RCI) was calculated. The RCI evaluates the reliable change by means of a variation of the standard error (SE) in the measurement which takes the pre-intervention and post-intervention evaluations into account. The change presented by an adolescent that is outside the range which could be attributed to the variability in measurement is considered the reliable change. Chi-square tests were performed. Finally, the moderating effects of gender, age, depression, anxiety and stress were tested using the PROCESS Macro for SPSS. The change from T1 to T2 in emotional intelligence, self-esteem, affect balance, difficulties, and prosocial behaviour were the dependent variables. The independent variable was the experimental condition.

## Results

The chi-square results indicated no statistically significant differences between the experimental group and the control group for gender (χ^2^ = 2.15; *p* = .143), age (χ^2^ = 6.71; *p* = .152), sexual orientation (χ^2^ = 6.71; *p* = .082), and family type (χ^2^ = 2.11; *p* = .716).

### MANOVA and MANCOVA

The results showed no statistically significant differences between the experimental group and the control group in the first evaluation (Wilks’ lambda, λ = .96, *F* = 0.48, *p* = .900; η^2^_p_ = .05). Both groups could therefore be considered comparable because they present similar baseline levels (Table 3). The MANCOVA results showed statistically significant differences between experimental group and control group after the implementation of the programme (Wilks’ λ = .77; *F* = 2.10; *p* = .035; η^2^_p_ = .22). Adolescents who participated in the programme showed more self-esteem and affect balance, and fewer emotional symptoms, behavioural problems, and hyperactivity than adolescents in the control group. The effect size on emotional intelligence, affect balance, peer and behavioural problems, hyperactivity, and prosocial behaviour ranges between η^2^_p_ = .01 and η^2^_p_ = .06; while the effect on self-esteem and emotional symptoms ranges between η^2^_p_ = .12 and η^2^_p_ = .15. Power analyses reveal that emotional intelligence and peer problems showed between 19% and 48% of statistical power; behavioural problems and hyperactivity showed between 70% and 78%; and self-esteem, emotional symptoms, and affective balance exceed 80% of statistical power (1-β).

**Table 3:** Impact of the programme comparing the experimental group and the control group.

		Experimental group	Control group	Cohen’s *d* ^a^ [95% CI] ^b^	ANOVA		ANCOVA		
		*M(SD)*	*M(SD)*		*F*	*p*	*F*	*p*	η^2^_p_^c^
AT	T1	24.18(5.99)	23.73(6.23)	0.07[-1.02, 1.17]	0.28	.597			
	T2	23.67(7.27)	22.53(7.00)	0.16[-1.17, 1.49]			0.35	.557	.01
CL	T1	24.82(6.56)	24.78(6.17)	0.01[-1.11, 1.13]	0.05	.822			
	T2	26.55(7.32)	24.91(6.57)	0.24[-1.04, 1.52]			1.57	.214	.02
RE	T1	27.32(7.24)	27.06(6.66)	0.04[-1.18, 1.26]	0.04	.835			
	T2	27.67(8.07)	26.74(6.11)	0.14[-1.13, 1.41]			0.20	.653	.01
SE	T1	30.03(5.88)	31.52(5.15)	-0.28[-1.24, 0.68]	1.79	.184			
	T2	31.03(6.82)	28.44(5.02)	0.47[-0.62, 1.55]			11.22	.001	.12
AB	T1	7.24(9.05)	8.35(6.67)	-0.15[-1.47, 1.17]	0.57	.453			
	T2	8.85(9.83)	7.16(8.15)	0.20[-1.44, 1.83]			5.50	.021	.06
ES	T1	3.76(2.26)	3.38(2.22)	0.17[-0.23, 0.57]	0.76	.386			
	T2	3.26(2.22)	4.49(2.61)	-0.50[-0.94, -0.05]			14.25	.000	.15
BP	T1	2.06(1.78)	2.14(1.62)	-0.05[-0.35, 0.25]	0.07	.793			
	T2	2.61(2.04)	3.16(2.36)	-0.25[-0.68, 0.19]			4.36	.040	.05
PP	T1	1.97(1.79)	1.41(1.57)	0.35[0.05, 0.64]	2.74	.101			
	T2	2.29(2.05)	2.76(2.22)	-0.22[-0.63, 0.20]			2.22	.140	.03
HY	T1	4.12(2.29)	4.16(2.38)	-0.02[-0.44, 0.41]	0.03	.867			
	T2	3.84(1.98)	4.49(2.16)	-0.31[-0.71, 0.09]			4.92	.029	.06
PB	T1	8.21(1.71)	8.33(1.36)	-0.08[-0.37, 0.22]	0.12	.733			
	T2	7.97(1.80)	7.66(1.94)	0.17[-0.20, 0.53]			0.19	.669	.01

AT, attention; CL, clarity; RE, repair; SE, self-esteem; AB, affect balance; ES, emotional symptoms; BP, behavioural problems; PP, peer problems; HY, hyperactivity; PB, prosocial behaviour.

^a^Effect size

^b^CI = confidence interval.

^c^Partial eta squared.

### Hierarchical multiple regression

The results of the first step in the hierarchical regression showed that the baseline levels of the adolescents influence changes in emotional intelligence, self-esteem, hedonic balance, prosocial behaviour, and total difficulties (Table 4). In other words, the higher the baseline scores, the less change (negative β). The second step showed that the experimental condition predicts change by controlling the baseline level of variables. The higher the condition (1>0), the greater the change from T1 to T2 (positive regression coefficient). In specific terms, the results indicated that adolescents in the experimental group had a greater change in self-esteem and affect balance (positive β), while their emotional problems and hyperactivity decreased (negative β).

**Table 4:** Hierarchical multiple regression analyses.

Outcome variables^a^	Regression: Model 1^b^				Regression: Model 2^c^				
	*ΔR*^*2*^ ^d^	*ΔF*^e^	ß^f^	*t*^g^	*ΔR*^*2*^ ^d^	*ΔF*^e^	ß^f^	*t*^g^	*DW*^h^
Attention	.14	17.37***	-.38	-4.17	< .01	0.44	-.06	0.66	2.13
Clarity	.16	20.07***	-.40	-4.48	.01	1.44	.11	1.20	2.19
Repair	.21	27.01***	-.45	-5.19	< .01	0.28	.05	0.53	1.87
Self-esteem	.16	17.43***	-.10	-4.18***	.10	11.57***	.31	3.40***	2.05
Affect balance	.04	4.30*	-.20	-2.07*	.04	4.34*	.20	2.08*	2.05
Emotional symptoms	.08	8.88**	-.30	-2.98**	.10	11.41***	-.32	-3.38***	2.16
Behavioural problems	.06	5.98*	-.25	-2.45*	.03	3.01	-.17	-1.73	2.04
Peer problems	.16	17.16***	-.40	-4.14***	.02	2.10	-.14	-1.45	1.99
Hyperactivity	.26	33.29***	-.51	-5.77***	.03	3.91*	-.17	-1.98*	2.22
Prosocial behaviour	.12	12.43***	-.34	-3.53***	.01	0.48	.07	0.69	1.61

^a^Outcome variables: change scores T1 to T2 were used for each for the regression analyses.

^b^Predictor = pre-intervention score

^c^Predictor = experimental condition, controlled for pre-intervention score

^d^Change in *R*^*2*^

^e^Change in *F*

^f^Regression coefficient

^g^Value of t-test statistic

^h^Durbin-Watson test.

*p≤.05.

**p≤.01.

***p≤.001.

### Reliable change index

The results show that the experimental group presented consistently higher or equal percentages of positive change and lower percentages of negative change than the control group (Table 5). The chi-square test show that only in behavioural problems the experimental group and the control group are statistically different (χ^2^ = 3.98; *p*≤.05).

**Table 5:** Reliable change index pre-intervention to post-intervention.

		Experimental Group			Control Group		
	χ^2^	RC^a^ *n*(*%*)	WRC^b^ *n*(*%*)	NRC^c^ *n*(*%*)	RC^a^ *n*(*%*)	WRC^b^ *n*(*%*)	NRC^c^ *n*(*%*)
Attention	0.29	5(15.2)	26(78.8)	2(6.1)	9(12.2)	59(79.7)	6(8.1)
Clarity	0.62	4(12.1)	27(81.8)	2(6.1)	8(10.8)	58(78.4)	8(10.8)
Repair	0.35	4(12.1)	26(78.8)	3(9.1)	7(9.5)	58(78.4)	9(12.2)
Self-esteem	4.77	2(6.7)	26(86.7)	2(6.7)	2(3.1)	46(71.9)	16(25.0)
Affect Balance	3.53	2(6.1)	29(87.9)	2(6.1)	3(4.1)	56(75.7)	15(20.3)
Emotional Symptoms	4.52	2(6.7)	28(93.3)	0(0.0)	1(1.5)	58(89.2)	6(9.2)
Behavioural problems	3.98*	0(0.0)	29(96.7)	1(3.3)	0(0.0)	53(81.5)	12(18.5)
Peer problems	2.74	0(0.0)	28(93.3)	2(6.7)	0(0.0)	52(80.0)	13(20.0)
Hyperactivity	1.72	1(3.3)	29(96.7)	0(0.0)	1(1.5)	61(93.8)	3(4.6)
Prosocial behaviour	2.27	0(0.0)	28(93.3)	2(6.7)	0(0.0)	53(81.5)	12(18.5)

^a^Reliable change

^b^Without reliable change

^c^Negative reliable change

*p≤.05.

### Moderating effects of gender, age, depression, anxiety, and stress

The moderating effects of gender, age, depression, anxiety, and stress on the impact of the intervention were tested (Table 6). Analyses indicate that anxiety moderated the influence of the intervention on self-esteem. Adolescents with low anxiety (*b* = 5.69; *t* = 4.09; *p*≤.001; LLCI = 2.93; ULCI = 8.46) and medium anxiety (*b* = 3.62; *t* = 3.71; *p*≤.001; LLCI = 1.68; ULCI = 5.56) improved their self-esteem with the intervention, while adolescents with high anxiety did not improve it.

**Table 6:** Moderating effects on the intervention programme.

T1–T2 change		Gender	Age	Depression	Anxiety	Stress
Attention	*b*^a^	.00	.01	.01	.01	.01
	*t*	0.35	-1.39	0.77	1.06	0.94
	95% CI	-3.99, 5.72	-7.84, 1.37	-0.22, 0.50	-0.15, 0.50	-0.17, 0.48
Clarity	*b*^a^	.00	.01	.00	.01	.02
	*t*	-0.25	-1.46	0.54	1.17	1.42
	95% CI	-6.21, 4.83	-7.22, 1.01	-0.27, 0.46	-0.12, 0.48	-0.10, 0.61
Repair	*b*^a^	.00	.00	.01	.00	.00
	*t*	0.13	0.75	-0.91	-0.40	-0.17
	95% CI	-5.20, 5.91	-2.88, 6.39	-0.55, 0.21	-0.45, 0.30	-0.39, 0.33
Self-esteem	*b*^a^	.01	.01	.02	.04	.01
	*t*	1.18	-0.63	-1.16	-2.55*	-1.01
	95% CI	-1.82, 7.14	-6.51, 3.39	-0.44, 0.11	-0.43, -0.05	-0.31, 0.10
Affect balance	*b*^a^	.00	.00	.00	.01	.00
	*t*	-0.73	-0.11	0.29	-0.92	0.70
	95% CI	-6.84, 3.16	-5.79, 5.19	-0.27, 0.37	-0.56, 0.21	-0.20, 0.42
Emotional symptoms	*b*^a^	.00	.00	.00	.00	.00
	*t*	0.58	-0.27	0.57	-0.22	0.47
	95% CI	-1.22, 2.23	-2.15, 1.64	-0.05, 0.10	-0.11, 0.09	-0.06, 0.09
Behavioural problems	*b*^a^	.00	.00	.00	.00	.00
	*t*	-0.33	-0.47	0.40	-0.68	-0.82
	95% CI	-1.94, 1.39	-1.51, 0.93	-0.06, 0.09	-0.13, 0.07	-0.12, 0.05
Peer problems	*b*^a^	.00	.00	.00	.00	.01
	*t*	-0.11	0.63	0.58	0.41	0.74
	95% CI	-1.83, 1.64	-1.16, 1.90	-0.08, 0.15	-0.07, 0.11	-0.08, 0.17
Hyperactivity	*b*^a^	.00	.00	.00	.01	.00
	*t*	0.34	0.43	-0.22	-0.95	0.44
	95% CI	-1.16, 1.64	-0.93, 1.45	-0.11, 0.09	-0.15, 0.05	-0.07, 0.10
Prosocial behaviour	*b*^a^	.00	.00	.00	.01	.00
	*t*	-0.29	0.88	0.23	-0.77	-0.17
	95% CI	-1.86, 1.38	-1.10, 1.29	-0.09, 0.11	-0.13, 0.06	-0.10, 0.09

^a^Refers to the unique contribution of the interaction term (moderator x group) to the prediction of the change score after controlling for the separate effects of group and moderator

*p≤.05.

## Discussion

The purpose of this study was to analyse the effectiveness of a game-based social-emotional programme on adolescents called *emoTIC*. Based on previous research related to social-emotional intervention programmes, our first hypothesis was that adolescents who participated in the game-based programme were expected to improve their emotional intelligence and self-esteem compared to a control group. The present study showed that *emoTIC* is effective to improve adolescents’ self-esteem, but not clearly their emotional intelligence. These results are partially in line with previous studies [27].

On the one hand, high emotional intelligence is composed of high clarity and repair as well as moderate attention [60]. Although attention tends to decline while clarity and repair tend to increase in the experimental group, the results of our study showed no statistically significant development. A previous mixed-model study evidenced that adolescents perceived an improvement of their emotional competences using *emoTIC* [58]. However, the improvement of emotional competences cannot be confirmed by the results of the present study. One possible explanation may be related to the measurement instrument used. Previous studies suggest that the instrument might not capture the changes with adequate sensitivity [61, 62].

On the other hand, the hypothesis is confirmed with respect to self-esteem. The adolescents who participated in the intervention programme increased their self-esteem; it means they improved their attitude towards themselves. Self-esteem is considered to be more of a dynamic rather than a static construct [63], and may therefore be incorporated into social-emotional programmes.

As for the second hypothesis, the adolescents who participated in the programme were expected to experience a positive effect on affect balance, emotional symptoms, behavioural problems, peer relationship problems, and hyperactivity [28, 39]. In overall terms, the results of the study support the hypothesis, and suggest that *emoTIC* may be an effective programme for preventing the increase of emotional, behavioural, and relational problems. The adolescents who completed the programme showed higher levels of affect balance and lower levels of emotional symptoms, behavioural problems, and hyperactivity compared with the control group. The activities carried out during the programme invited the adolescents to focus on positive aspects of their lives and to reflect on their emotions, which may have influenced their hedonic balance and emotional symptoms. Moreover, the dynamism of the group sessions, combined with the use of smartphones, may have helped the adolescents to focus their attention on activities and enabled positive interaction with their peers, which may have influenced their hyperactivity, and their peer and behavioural problems.

Finally, previous studies suggest that demographic factors and affective symptoms may moderate the effect of the intervention programmes [42]. The third hypothesis therefore tested a model of moderation with gender, age, depression, anxiety, and stress as the moderating variables. The results obtained suggest that anxiety may moderate the effectiveness of the programme in improving self-esteem. Adolescents with low or medium anxiety benefited from the programme, while adolescents with high anxiety did not improve their self-esteem. It should therefore be noted that these adolescents may not fully benefit from *emoTIC*. Knowledge about the potential moderators of the programme effectiveness is essential for increasing the effectiveness of future intervention programmes [42].

This research has several strengths. The effectiveness of an intervention programme implemented through technologies to develop social-emotional competences has been assessed in adolescents. Triangulation of the data was performed using MANCOVA, multiple regression analysis and RCI to determine the effectiveness of *emoTIC*. The moderating effects were also tested. Despite its strengths, the present study has some limitations. First, only self-report measures were included, and it would be advisable to introduce performance measures for the assessment of emotional competences. Second, it would be useful to include an additional measure to determine the effects of the programme in the long term. Third, some potential covariables (e.g. IQ, socio-economic status, previous or history of mental disorders) were not taken into account should be assessed in future research. Four, a random sampling method should have been used instead of non-probability sampling such as convenience sampling. Likewise, the sample size should be increased in future research.

In conclusion, this study examines *emoTIC*, a game-based social-emotional programme that influences personal, emotional, and social factors during adolescence. Specifically, *emoTIC* improve self-esteem, an essential moderator of the relationship between emotional and behavioural problems, and suicidal behaviour. Adolescents with emotional and behavioural difficulties show lower risk of committing suicidal behaviours when they have high self-esteem [47]. This game-based programme may therefore protect adolescents’ mental health. In the same way, affect balance, the affective dimension of subjective well-being [48], may be enhanced by *emoTIC*. Finally, this game-based programme may decrease emotional symptoms, behavioural problems and hyperactivity of adolescents. During this developmental stage, adolescents are sensitive to the emerging of emotional instability symptoms [18], and programmes such as *emoTIC* can provide adolescents with skills to manage their own emotional states. This programme has a double impact. On the one hand, the intervention programme enhances strengths that protect adolescents’ development. On the other hand, it reduces risk factors that can lead to the emergence of mental health problems. With regard to the implementation, the development of emotional intelligence by means of technologies could be more attractive and useful for adolescents, and could increase their commitment to the programme [35, 36]. Moreover, the approach of *emoTIC* as a game adventure may generate positive and pleasant emotions in adolescents, which may facilitate learning and contribute to academic achievement [5]. Using technology in the development of social-emotional programmes could be the first step towards increasing interest in emotions in adolescents.

## Supporting information

S1 Dataset (XLSX)
